# Biocatalysis of d,l-Peptide Nanofibrillar Hydrogel

**DOI:** 10.3390/molecules25132995

**Published:** 2020-06-30

**Authors:** Tiziano Carlomagno, Maria C. Cringoli, Slavko Kralj, Marina Kurbasic, Paolo Fornasiero, Paolo Pengo, Silvia Marchesan

**Affiliations:** 1 Chemical & Pharmaceutical Sciences Department, University of Trieste, 34127 Trieste, Italy; tiziano.carlomagno@studenti.units.it (T.C.); mcringoli@units.it (M.C.C.); marina.kurbasic@phd.units.it (M.K.); pfornasiero@units.it (P.F.); 2 INSTM Trieste Research Unit, 34127 Trieste, Italy; 3 Materials Synthesis Department, Jožef Stefan Institute, 1000 Ljubljana, Slovenia; slavko.kralj@ijs.si; 4 ICCOM-CNR Trieste Research Unit, 34127 Trieste, Italy

**Keywords:** biocatalysis, fibrils, hydrogel, self-assembly, supramolecular material, peptide, amyloid, histidine, D-amino acids, esterase

## Abstract

Self-assembling peptides are attracting wide interest as biodegradable building blocks to achieve functional nanomaterials that do not persist in the environment. Amongst the many applications, biocatalysis is gaining momentum, although a clear structure-to-activity relationship is still lacking. This work applied emerging design rules to the heterochiral octapeptide sequence His–Leu–^D^Leu–Ile–His–Leu–^D^Leu–Ile for self-assembly into nanofibrils that, at higher concentration, give rise to a supramolecular hydrogel for the mimicry of esterase-like activity. The peptide was synthesized by solid-phase and purified by HPLC, while its identity was confirmed by ^1^H-NMR and electrospray ionization (ESI)-MS. The hydrogel formed by this peptide was studied with oscillatory rheometry, and the supramolecular behavior of the peptide was investigated with transmission electron microscopy (TEM) analysis, circular dichroism (CD) spectroscopy, thioflavin T amyloid fluorescence assay, and attenuated total reflectance (ATR) Fourier-transform infrared (FT-IR) spectroscopy. The biocatalytic activity was studied by monitoring the hydrolysis of *p*-nitrophenyl acetate (pNPA) at neutral pH, and the reaction kinetics followed an apparent Michaelis–Menten model, for which a Lineweaver–Burk plot was produced to determine its enzymatic parameters for a comparison with the literature. Finally, LC–MS analysis was conducted on a series of experiments to evaluate the extent of, if any, undesired peptide acetylation at the N-terminus. In conclusion, we provide new insights that allow gaining a clearer picture of self-assembling peptide design rules for biocatalysis.

## 1. Introduction

Enzymes have long been a great source of inspiration for organic chemists to develop new catalysts for industrial applications, as well as to achieve simple, low-cost mimetics that display activity in water and that are biodegradable, so that they do not persist in the environment [1]. One of the simplest approaches consists of the use of short peptides, since it is possible to directly incorporate the specific residues that perform the enzymatic catalysis in the amino-acid sequence. Self-assembling peptides offer additional advantages, such as the possibility to create hydrophobic pockets that provide ideal environments for reactions to occur, as well as multivalency for improved performance, thanks to the spatial proximity of multiple functional groups. It is, thus, not surprising that the topic is widely reviewed [2,3,4,5].

In particular, hydrolases are a class of enzymes that are of high relevance for industrial applications and for organic synthesis; thus, they provide a good target for biomimicry. They include enzymes such as lipases and proteases, which are both widely studied and used. Typically, a biomimetic sequence contains a histidine (His) residue that is a common feature in the catalytic active site. Prins et al. developed elegant systems, whereby a short His-containing peptide self-assembled on the surface of gold nanoparticles, yielding efficient transesterification catalysis [6]. Subsequent studies confirmed that self-assembly is a prerequisite to achieve catalytic efficiency, which can be enhanced by the presence of serine (Ser) residues [7]. Furthermore, different peptides can be used to increase the system complexity and achieve a dynamic structure [8]. Divalent cations can also be useful additives to promote the activity of hydrolytic metallo-nanozymes [9]. Indeed, typical approaches to activate the His residue toward catalysis comprise either inclusion of Ser and/or aspartate (Asp) to reconstitute the hydrolase catalytic triad as described by Luisi et al. and many others, such as Ulijn and Matsui [10,11], as well as Guler et al. [12], or metal coordination usually featuring divalent zinc, as shown for instance in an elegant study by DeGrado and Korendovych [13]. Other ions were studied by Hamley and Banerjee, such as trivalent iron or divalent mercury [14]. Arginine (Arg) also proved to be a useful inclusion to enhance catalytic activity when featured on supramolecular systems based on peptides developed by Liu et al. [15,16]. As knowledge of supramolecular peptide biocatalysts increased, amino-acid sequences could be progressively shortened in a minimalistic approach down to, remarkably, the single amino acid phenylalanine when self-assembled in the presence of zinc ions as recently reported by Gazit et al. [17].

However, at present, it is still not possible to predict exactly the performance of a de novo designed peptide. Amongst the various approaches to achieve a self-assembling short peptide, an attractive strategy features the inclusion of both D- and L-amino acids at specific positions along a heterochiral sequence, whereby nanostructured hydrogels can be formed even with just three amino acids, while their homochiral isomers typically precipitate in water [18]. These supramolecular systems feature amphipathic assemblies, whereby the different stereoconfiguration allows displaying hydrophobic side chains on the same side of the peptide backbone [19,20]. This design was also successfully applied to His–^D^Phe–^D^Phe, which displayed biocatalytic activity only in the self-assembled form, although it formed hydrogels at the very high concentration of 50 mM, thus limiting its practical use [21]. To validate the design on longer and more diverse heterochiral sequences to achieve nanostructured functional materials, we designed the octapeptide His–Leu–^D^Leu–Ile–His–Leu–^D^Leu–Ile for this study. The sequence is devoid of phenylalanine, which is featured in all other gelling heterochiral unprotected peptides [18], to expand the sequence design. It is based on a tetramer sequence that features His as a catalytic site, as well as hydrophobic residues leucine (Leu) and isoleucine (Ile) that are expected to provide sufficient hydrophobicity for self-assembly in aqueous solutions. The choice for an octapeptide allows for a more appropriate comparison with other biocatalysts that feature two His residues and a similar number of amino acids [13,15,22].

## 2. Results and Discussion

### 2.1. Peptide Self-Assembly

**Scheme 1:**
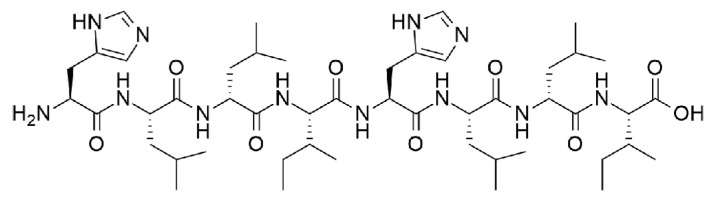
Chemical structure of His–Leu–^D^Leu–Ile–His–Leu–^D^Leu–Ile.

His–Leu–^D^Leu–Ile–His–Leu–^D^Leu–Ile (Scheme 1) was synthesized in solid phase and purified by reverse-phase HPLC, following published procedures [20]. Peptide identity and purity was confirmed by ^1^H-NMR and electrospray ionization (ESI)-MS (see Supplementary Materials). Its hydrophobicity was evident in its limited water solubility at neutral pH. Dissolution was possible at acidic pH, whereby the peptide is present in its cationic form. A pH change to neutral was then used to trigger fibrillization, which, at higher concentrations, led to the formation of self-supportive hydrogels, with a minimum gelling concentration (mgc) of 10 mM. The viscoelastic properties of the hydrogel were assessed by oscillatory rheometry (see Supplementary Materials). At mgc, the peptide gelled immediately and reached a G′ of 1 kPa, which remained stable over time. Frequency sweeps confirmed G′ > G″, with both values being independent of applied frequency, as expected for a stable hydrogel. Stress sweeps confirmed a linear viscoelastic region until 1 Pa, with a gel-to-sol transition occurring above 10 Pa.

**Figure 1:**
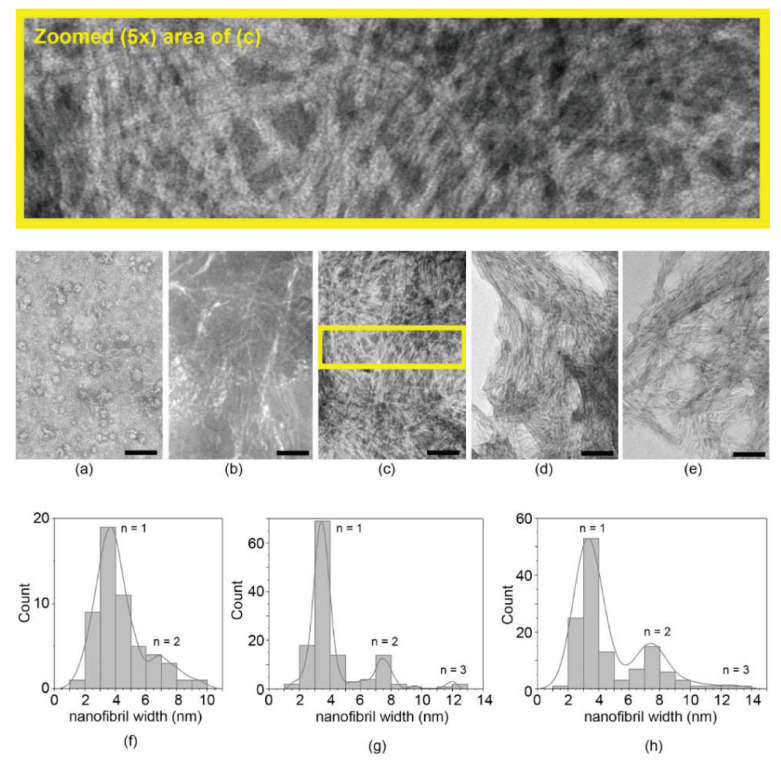
TEM analysis of peptide samples at various concentrations: (**a**) 0.05 mM; (**b**) 0.1 mM; (**c**) 1 mM; (**d**) 5 mM; (**e**) 10 mM. The yellow rectangle in (**c**) corresponds to the zoomed area (5×) shown above to assist the reader with fibril visualization. Nanofibril size distribution revealed consistent data over the concentrations tested, suggesting nanofibrils of approximately 3.5 nm width (*n* = 1), occasionally bundling in pairs (*n* = 2) or, rarely, in triplets (*n* = 3): (**f**) 0.1 mM (*n* = 50); (**g**) 1 mM (*n* = 130); (**h**) 10 mM (*n* = 130). Scale bar = 200 nm in (**a**), and 100 nm in (**b**–**e**).

Transmission electron microscopy (TEM) analysis was, thus, performed to analyze the nanomorphology leading to the hydrogel at mgc, as well as the presence of any nanostructure at lower concentrations (Figure 1). At 0.05 mM, only rare instances of short nanofibrils were noted, and the sample was mainly characterized by spherical morphologies suggesting peptide nucleation, which was likely driven by hydrophobicity (Figure 1a). At 0.1 mM, nanofibrils became evident, albeit spanning only a few hundred nanometers in length (Figure 1b). At concentrations ≥1 mM (Figure 1c–e), samples revealed dense networks of homogeneously sized fibrils with length spanning several microns. Fibril diameter (3.5 nm) was unchanged across samples; occasionally, bundles of two, and rarely of three, nanofibrils were noted, with no significant difference across the concentrations tested (Figure 1f–h). Contrarily to other heterochiral amyloid peptides, no increase in hierarchical bundling was noted with increasing concentration, suggesting that the main difference between samples at 1, 5, or 10 mM simply concerned the number of fibrils [19,20,21].

### 2.2. Peptide Secondary Structure

**Figure 2:**
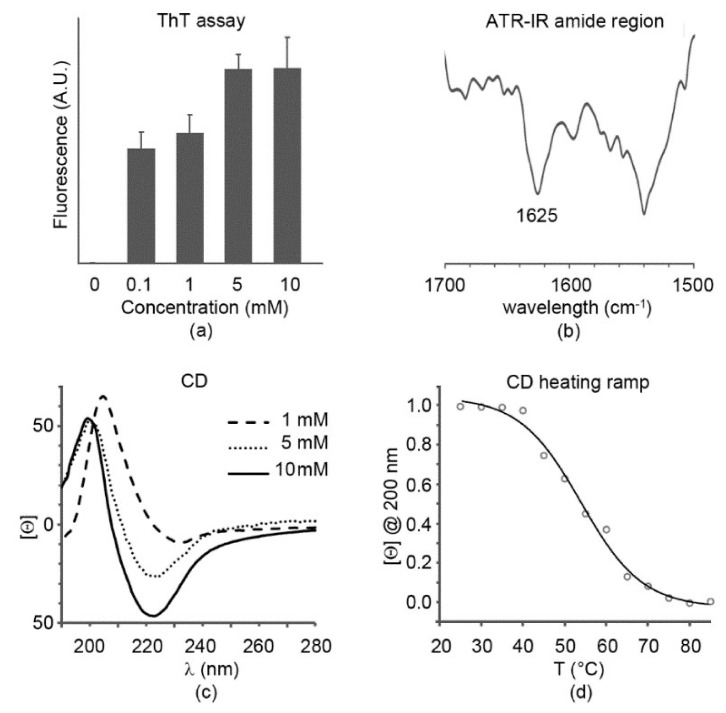
Peptide secondary structure. (**a**) Thioflavin T fluorescence assay; (**b**) attenuated total reflectance infrared (ATR-IR) amide region at 10 mM; (**c**) circular dichroism (CD) spectra; (**d**) CD heating ramp revealing a T_m_ = 54 °C at 10 mM.

TEM analysis suggested an amyloid nature for the octapeptide; therefore, thioflavin T was used to confirm this hypothesis. This is a dye that was shown to bind to β-sheet amyloid fibril surfaces; as a result of this interaction, the rotation between the benzothiazole and the benzene rings is impeded, leading to fluorescence [23]. Already at 0.1 mM, significant fluorescence was noted, with a non-linear increase at higher concentrations, as typically shown for amyloids (Figure 2a). Fluorescence appeared to plateau at the highest concentration tested, 10 mM, which corresponded to the mgc.

Attenuated total reflectance (ATR) infrared spectra displayed in the amide region a main signal at 1625 cm^−1^ that is diagnostic for amyloids (Figure 2b). Another major signal at 1540 cm^−1^ was due to the His imidazole ring [24], as well as the peak at 1568 cm^−1^. Furthermore, a minor peak at 1694 cm^−1^ suggested presence of the C-terminal carboxylic group in its neutral form [25], as already reported for other amyloids, whereby the supramolecular organization disfavored deprotonation [26,27,28,29,30,31].

Circular dichroism (CD) spectroscopy also confirmed a signature compatible with β-sheets, with a minimum at 225 nm and a maximum at 200 nm at the mgc (Figure 2c). The spectrum was similar at 5 mM, in agreement with the fluorescence data, although with a less intense β-sheet minimum. Lowering the concentration further to 1 mM led to red-shifts at both peaks. Heating ramps from room temperature up to 85 °C allowed monitoring the loss of the supramolecular structure, with a T_m_ of 54 °C (Figure 2d), thus indicating that the gel at mgc displayed good stability.

### 2.3. Biocatalytic Performance

The esterase-like activity of the supramolecular structures was evaluated by monitoring the hydrolysis of 4-nitrophenyl acetate (pNPA). The product formed was 4-nitrophenol (pNP) that displayed a maximum of absorbance at 405 nm. The catalysis was performed at different concentrations of peptide, 100 μM, 1 mM, 5 mM, and 10 mM (mgc) at pH 7.0. Initial velocities (V_i_) for pNPA hydrolysis without catalyst (see Supplementary Materials) were subtracted from all samples. The absorbance curves fitted well as first-order kinetics, with the initial velocities (V_i_) being directly proportional to pNPA concentration (Table 1 and Supplementary Materials).

**Figure 3:**
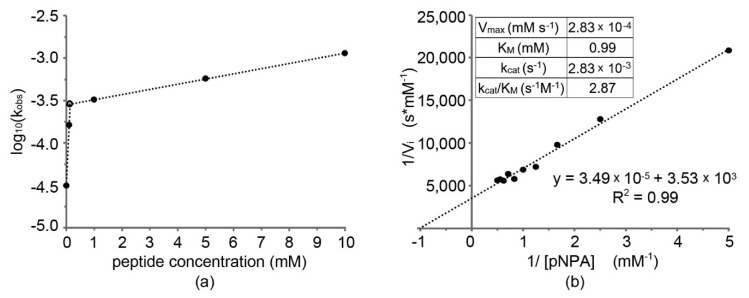
(**a**) Log_10_(k_obs_) against peptide concentration; (**b**) Lineweaver–Burk plot with catalytic parameters (inset).

Figure 3a shows the trend of the apparent activation free energy (ΔG^‡^) of the reaction, which is proportional to log(k_obs_). Catalysis occurred in the presence of supramolecular structures formed by peptide self-assembly.

From the significant increase of k_obs_ already at low concentrations of peptide, we inferred that amyloid nanofibrils, which were the active catalyst species, formed in sufficient quantity to significantly catalyze the reaction at a concentration of ~0.1 mM (intersection point in Figure 3a), confirming the results obtained by TEM analysis. At higher concentrations, a further increase of k_obs_ was less dramatic, and it was mostly pertinent to the increase in surface area of nanofibrils present that increased with the concentration as shown by TEM micrographs. It is worth noting that catalytic tests performed with 10 mM peptide were carried out in the hydrogel state, yet no evident detrimental effect due to limited diffusion was noted on the catalytic performance. We inferred that the soft nature of the hydrogel, having a G′ of 1 kPa at the mgc of 10 mM, permitted the fast diffusion of small molecules throughout the sample.

To characterize this peptide with its catalytic parameters according to the Michaelis–Menten enzymatic model, it was necessary to collect a series of data points for substrate concentration significantly greater than catalyst concentration. Moreover, it was not possible to use high concentrations of pNPA, due to the saturation of the signal in short times and to dissolution problems in MeOH. For this reason, 0.1 mM was a peptide concentration suitable for this type of analysis. The data obtained, reported in the inset in Figure 3b, fitted well an apparent Michaelis–Menten function, and a Lineweaver–Burk plot was realized (Figure 3b) to extrapolate the value of maximum velocity (V_max_) and the Michaelis–Menten constant (K_M_) and, thus, calculate the turnover number (k_cat_). Finally, the ratio k_cat_/K_M_, which is an evaluation of the capacity of the peptide to catalyze this specific reaction, was obtained for a comparison against literature data (Table 2). For a meaningful comparison, peptide catalysts activated with zinc cations or with catalytic parameters derived at a very different pH were not included. Overall, the designed catalyst is within range of reported self-assembling peptide performance for ester hydrolysis, although it is clearly surpassed by the peptide amphiphile reported by Guler and Stupp [22]. Other peptides, of similar length to the one of this study (7-mers), also showed very efficient performance; however, their catalytic parameters were assessed under different conditions, i.e., with zinc dication as an activator, and at pH 8.0 [13].

The free amino group at the N-terminus of the peptide could potentially act as a nucleophile, leading to peptide acetylation. To verify this hypothesis, further experiments were carried out at different concentrations of peptide and substrate, and the reactions were analyzed by LC–MS. The ratio of concentrations investigated went from an excess of peptide to an excess of substrate, and the percentage of pNPA converted by nucleophilic acyl substitution (S_N_Ac) was calculated and is reported in Table 3. In all cases, regardless of the conditions tested, the vast majority of pNPA underwent the desired catalyzed hydrolysis, rather than S_N_Ac. From an analysis of the percentages of pNPA hydrolyzed by nucleophilic acyl substitution, it is possible to notice that, upon increasing [pNPA] compared to the peptide, there was a decrease in the quantity of substrate converted by nucleophilic attack. This was due to the reduction of probability to collide and react with the amino group. Consequently, the amount of pNPA hydrolyzed by water, with peptide-mediated catalysis, increased. On the other hand, at higher concentrations of peptide, the quantity of pNPA that reacted with the amino group was greater, because of the increased competitiveness of the acetylation compared to hydrolysis. A potential strategy to address this issue could be peptide cyclization or acetylation. Unfortunately, at high amounts of acetylated peptide, insoluble aggregates were noted that required dissolution by dimethyl sulfoxide (DMSO) prior to LC–MS analysis. This observation suggested that the octapeptide acetylation disrupted self-assembly. Therefore, other design strategies, other than acetylation, will be needed to enhance these systems.

In conclusion, this work described a nanostructured hydrogel in phosphate buffer of an octapeptide that displayed esterase-like catalytic activity in the assembled state. Peptide design based on heterochirality was successful, showing that the emerging rules for the correct positioning of D-amino acids along hydrophobic short sequences can be extended beyond tripeptides [20]. Nanofibrils were formed to a large extent already at 0.1 mM, while the minimum gelling concentration corresponded to 10 mM. Catalytic activity followed Michaelis–Menten kinetics, and the performance was within range of reported short peptides without zinc activation, which is relevant to phosphate-buffered systems that are incompatible with zinc, as zinc phosphate would precipitate out of solution. Overall, this study expanded our understanding of this kind of peptide catalyst, while further improvements will be needed to achieve very efficient catalysts with this design.

## 3. Materials and Methods

### 3.1. Materials

2-Chlorotrityl resin, *O*-benzotriazole-*N,N,N,N′*-tetramethyl-uronium-hexafluoro-phosphate (HBTU), and Fmoc-protected amino acids were purchased from GL Biochem (Shanghai, China) Ltd. All solvents were purchased of analytical grade from Merck. Piperidine, trifluoroacetic acid (TFA), *N,N*-diisopropyl ethyl amine (DIPEA), and triisopropyl silane (TIPS) were from Acros (Milan, Italy). Sodium dihydrogen phosphate and disodium hydrogen phosphate were from BDH AnalaR (Milan, Italy). High-purity Milli-Q-water (MQ water) with a resistivity greater than 18 MΩ·cm was obtained from an in-line Millipore RiOs/Origin system.

### 3.2. Peptide Synthesis and Characterisation

The peptide was synthesized in solid phase on chlorotrityl resin, using Fmoc protection and HBTU activation, followed by purification using reverse-phase HPLC, using standard procedures [20]. ^1^H-NMR spectra were recorded at 400 MHz on a Varian Innova Instrument with chemical shift reported as ppm (in DMSO or MeCN with tetramethylsilane as internal standard). ESI-MS spectra were recorded on an Agilent 6120 single quadrupole LC–MS system.

### 3.3. Self-Assembly Protocol

The peptide was dissolved in a sodium dihydrogen phosphate solution (0.2 M) and brought to pH 2.0 with the addition of 1 M HCl (to reach overall 85% of final volume), as well as with the aid of sonication in an ultrasound bath (Branson, Milan, Italy). Self-assembly was triggered by the addition of 1 M NaOH to achieve the desired final concentration at pH 7.0.

### 3.4. Oscillatory Rheometry

Dynamic time sweep rheological analysis was conducted on a Malvern Kinexus Ultra Plus Rheometer (Alfatest, Milan, Italy) with a 20-mm stainless-steel parallel plate geometry. The temperature was maintained at 25 °C using a Peltier temperature controller. Samples were prepared in situ and immediately analyzed with a gap of 1 mm. Time sweeps were recorded using a frequency of 1 Hz and a controlled stress of 0.50 Pa. Frequency sweeps were recorded using a controlled stress of 0.50 Pa, and then stress sweeps were recorded using a frequency of 1 Hz.

### 3.5. Transmission Electron Microscopy (TEM)

The nanostructure morphology of the peptide was determined at 50 μM, 100 μM, 1 mM, 5 mM, and 10 mM with a transmission electron microscope (TEM). Firstly, 5 μL of the peptide solution/hydrogel was poured on copper-lacey carbon-coated 300-mesh grids, while a TEM grid was exposed for 6 min under ultraviolet (UV) ozone cleaner just before material deposition. After 1 min of adsorption, the excess was drawn off, and 5 μL of a 2% aqueous potassium phosphotungstate at pH 7.2 was poured on the grids. Grids were air-dried until needed, and TEM images were acquired using a Jeol JEM 2100 instrument at 100 kV. Images were analyzed using the free software ImageJ (Wayne Rasband, Madison, FIJI), and the measurements were performed manually (*n* = 130 for 1 mM and 10 mM samples; *n* = 50 for 0.1 mM sample because fibrils were fewer due to the significantly lower peptide concentration).

### 3.6. Thioflavin T Fluorescence

Fluorescence emission was measured with an Infinite M1000 Pro TECAN plate reader, using an excitation wavelength of 446 nm and an emission wavelength of 484 nm. Firstly, 120 μL of the peptide solutions and 30 μL of ThT solution (24 μM in 20 mM glycine-NaOH pH 7.5, filtered with a 0.2-μm filter) were poured in a 96-U bottom black polystyrene microplate, and fluorescence was measured after 15 min.

### 3.7. ATR-IR Spectroscopy

The infrared (IR) spectra were recorded with a Jasco 4700 Fourier-transform (FT)-IR, equipped with an ATR Pro One. A drop of the hydrogel was placed on a silicon wafer, and then dried under vacuum. Spectra were acquired with 1-cm^−1^ resolution.

### 3.8. Circular Dichroism (CD) Spectroscopy

A 0.1-mm quartz cell was used on a Jasco J815 Spectropolarimeter, with 1-s integrations, one accumulation, and a step size of 1 nm with a bandwidth of 1 nm over a range of wavelengths from 190 to 280 nm at 20 °C. Samples were prepared as indicated above in Section 3.3 in the CD cell, and spectra were immediately recorded. Shown spectra are the average of at least five measurements. Heating ramps were recorded from 25 °C to 85 °C, with 5 °C intervals.

### 3.9. Biocatalysis

The esterase activity of the peptide was evaluated by following the hydrolysis of 4-nitrophenyl acetate (pNPA). The formation of the product was analyzed with an Infinite M1000 Pro TECAN plate reader at a wavelength of 405 nm. The reaction was monitored at 10 mM, 5 mM, 1 mM, and 0.1 mM of peptide and 1.0 mM, 0.8 mM, 0.6 mM, 0.4 mM, and 0.2 mM of pNPA (*n* = 6 for each condition). In each well, 90 μL of peptide solution at pH 7.0 and 10 μL of pNPA in MeOH were added. A Michaelis–Menten plot and a Lineweaver–Burk plot were realized using a concentration of pNPA from 0.2 mM to 2 mM and 0.1 mM of peptide.

## Figures and Tables

**Table 1:** Initial velocities (V_i_ expressed in mM/s) at different concentrations of 4-nitrophenyl acetate (pNPA) (0.2–1.0 mM) and different concentrations of peptide catalyst (0.1, 1, 5, and 10 mM).

[pNPA]	0.1 mM	1 mM	5 mM	10 mM
0.2	6.25 × 10^−6^	7.14 × 10^−5^	1.10 × 10^−4^	2.24 × 10^−4^
0.4	1.26 × 10^−5^	1.31 × 10^−4^	1.85 × 10^−4^	4.32 × 10^−4^
0.6	1.87 × 10^−5^	1.76 × 10^−4^	4.48 × 10^−4^	7.40 × 10^−4^
0.8	2.51 × 10^−5^	2.60 × 10^−4^	4.07 × 10^−4^	9.30 × 10^−4^
1.0	3.13 × 10^−5^	3.29 × 10^−4^	5.68 × 10^−4^	1.11 × 10^−3^
k_obs_	1.63 × 10^−4^	3.22 × 10^−4^	5.72 × 10^−4^	1.14 × 10^−3^

**Table 2:** Comparison of catalytic performance with literature data on short peptides without Zn^2+^.

Peptide Sequence	Number of His	pH	k_cat_/K_M_ (s^−1^M^−1^)	Reference
cyclo(HF) + cyclo(CF)	1	7.5	0.09	[32]
HSGQQKFQFQFEQQ	1	7.4	0.09	[15]
cyclo(HF) + cyclo(CV)	1	7.3	0.13	[32]
HSGQQKFQFQFEQQ + RSGQQKFQFQFEQQ	1	7.4	0.15	[15]
cyclo(HF) + cyclo(CL)	1	7.4	0.2	[32]
HL^D^LIHL^D^LI	2	7.0	2.9	This work
H_2_KLLLAAA(Nε-palmytoyl)K-NH_2_	2	7.4	20	[22]

**Table 3:** LC–MS analysis to verify the extent of amine-mediated acetylation

[Peptide] (mM)	[pNPA] (mM)	% Ac-Peptide (S_N_Ac)	% pNPA (S_N_Ac)	% pNPA (Catalysis)
1.0	0.2	7	35	65
1.0	1.0	26	26	74
0.1	0.4	36	9	91
0.1	1.0	64	6	94
0.1	2.0	81	4	96

## Supplementary Materials

The following are available online: spectroscopic data, rheometry data, biocatalysis data.
