# Supplementary material for: Biocatalysis of d,l-Peptide Nanofibrillar Hydrogel
Source: Molecules. 2020 Jun 30;25(13):2995. doi: 10.3390/molecules25132995 (PMC7411710; doi:10.3390/molecules25132995)
Supplement: Supplementary file 1 [file molecules-25-02995-s001.pdf]

Supporting Information

Biocatalysis of D,L-peptide nanofibrillar hydrogel

Tiziano Carlomagno [1], Maria C. Cringoli[1,2], Slavko Kralj[3], Marina Kurbasic[1], Paolo Fornasiero[1,2,4], Paolo Pengo[1*], and Silvia Marchesan[1,2*]

[1] University of Trieste, Chemical & Pharmaceutical Sciences Department, Italy
[2] INSTM Trieste Research Unit, Italy
[3] Jožef Stefan Institute, Materials Synthesis Department, Ljubljana, Slovenia
[4] ICCOM-CNR Trieste Research Unit
* Correspondence: ppengo@units.it; smarchesan@units.it.

Table of Contents

1. Peptide spectroscopic data………………………………………………………………… 2

2. Rheometry data……………………………………………………………………………… 5

3. Biocatalysis data…………………………………………………………………………….. 6

1. Peptide spectroscopic data

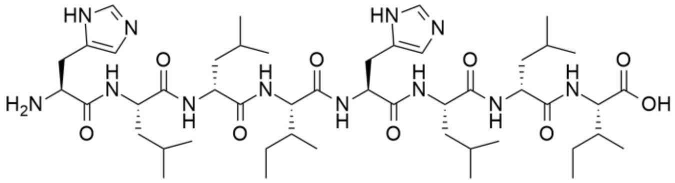

^{1}H-NMR (400 MHz, DMSO-d_6) δ (ppm): 8.70 (s, 2H, HimCH His), 8.62 (d, 1H, NH), 8.41 (d, 1H, NH), 8.29 (d, 1H, NH), 8.03 (d, 1H, NH), 7.99 (d, 1H, NH), 7.96 (d, 1H, NH), 4.56 (dd, 1H, CH His), 4,38 (m, 4H, CH Leu), 4.18 (m, 2H, CH Ile), 4.04 (dd, 1H, CH His), 3.01 (m, 2H, CH_2 His), 3.01 (dd, 1H, CH_2 His), 2.86 (dd, 1H, CH_2 His), 1.80-1.30 (m, 16H, CH, CH_2), 1.21-1.01 (m, 2H, CH), 0.88-0.73 (m, 36H, CH_3). **MS (ESI):** m/z 972.5 $(M+H)^+$ 486.4 $(M+2H)^{2+}$, $C_{46}H_xN_{12}O_x$ requires 971.2.

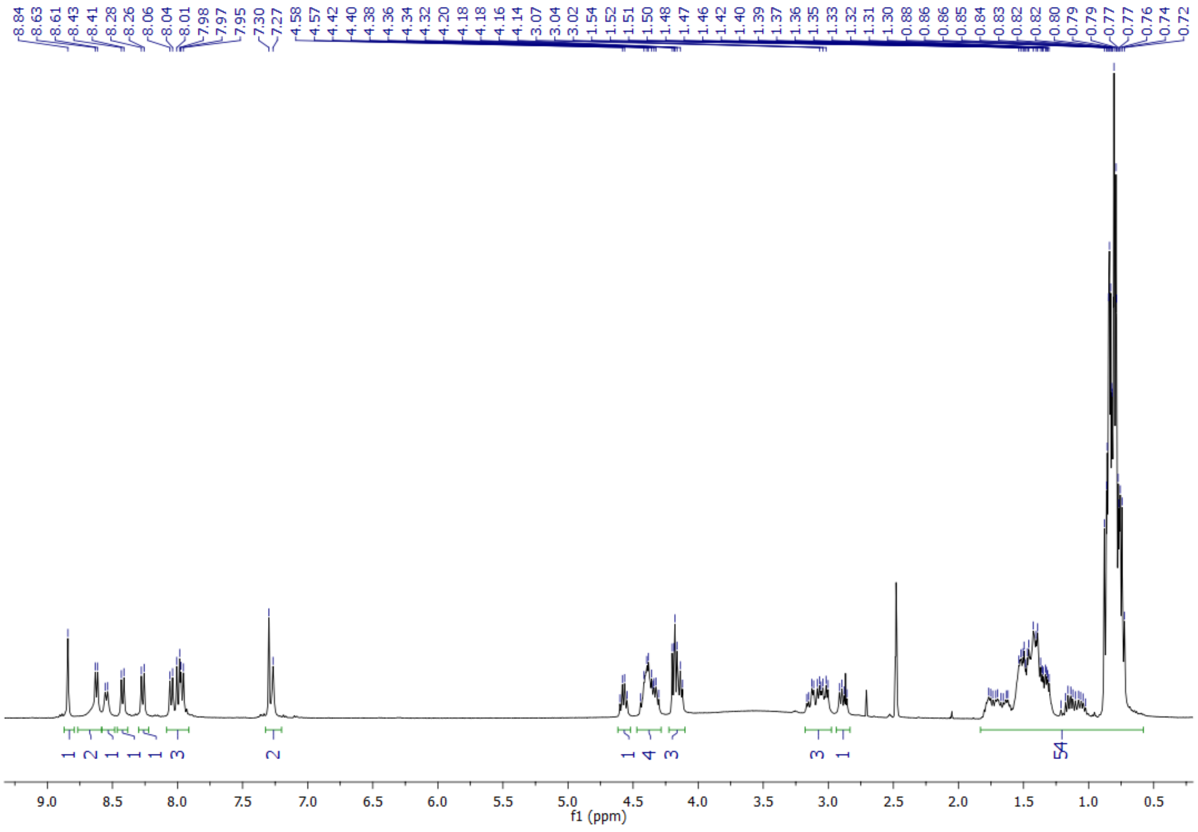

Fig. S1. ^{1}H-NMR spectrum of the octapeptide.

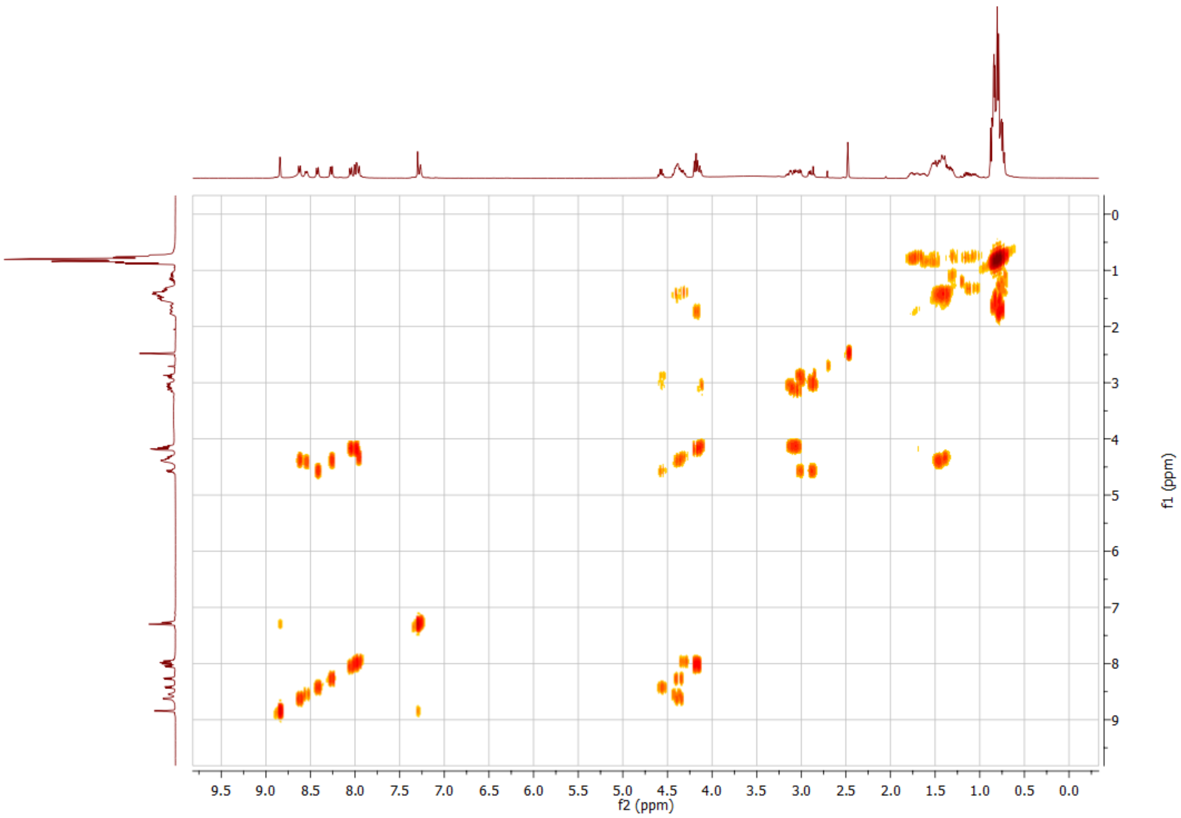

Fig. S2. COSY spectrum of the octapeptide.

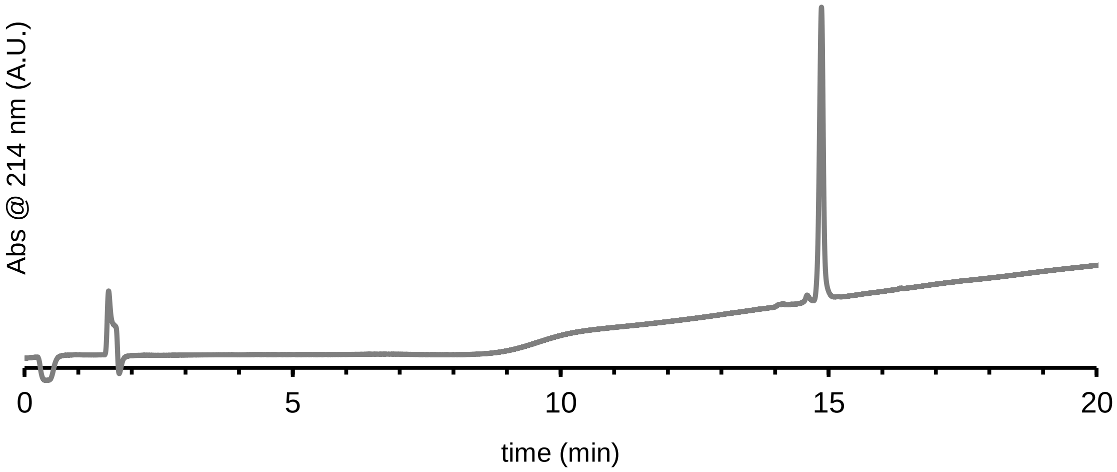

Fig. S3. HPLC trace of the purified octapeptide.

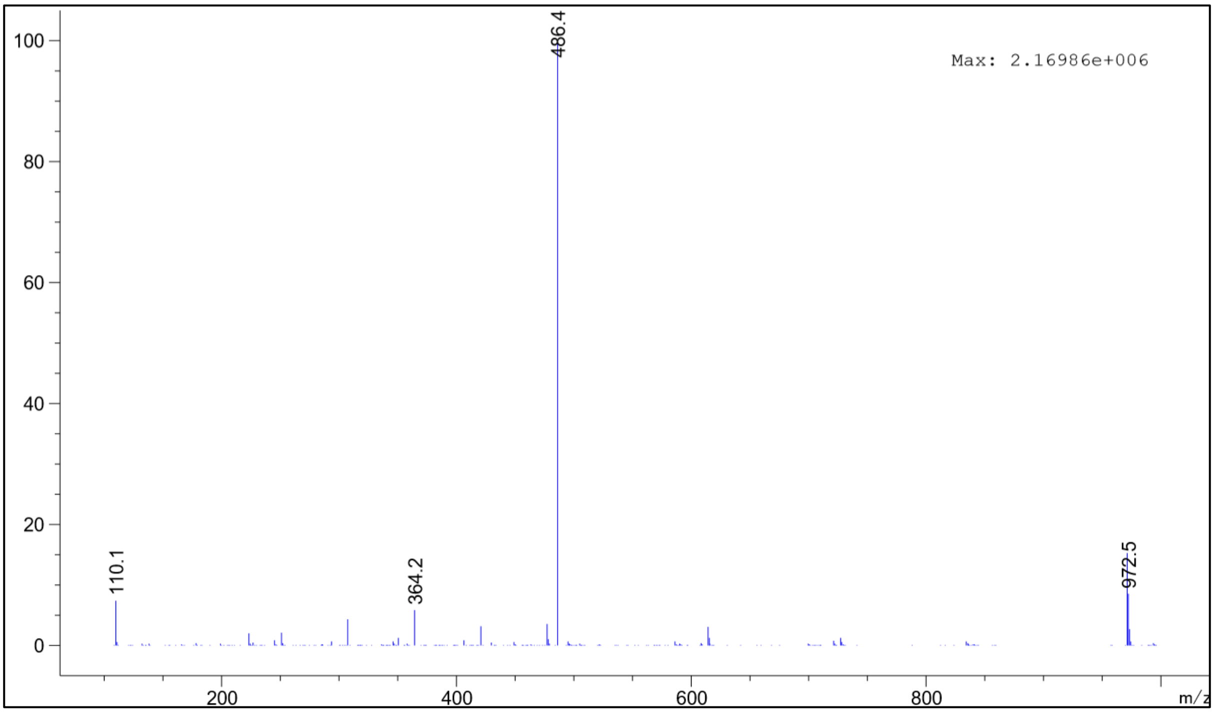

Fig. S4. ESI-MS spectrum of the octapeptide (positive ion mode).

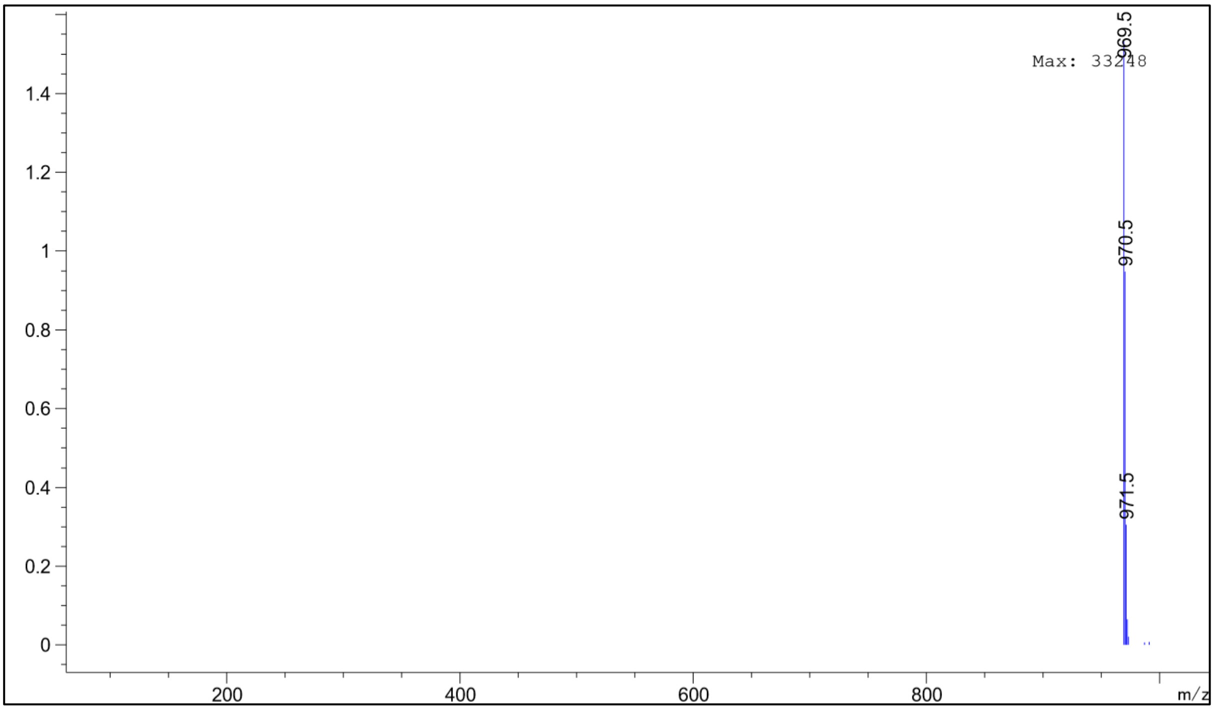

Fig. S5. ESI-MS spectrum of the octapeptide (negative ion mode).

2. Rheometry data

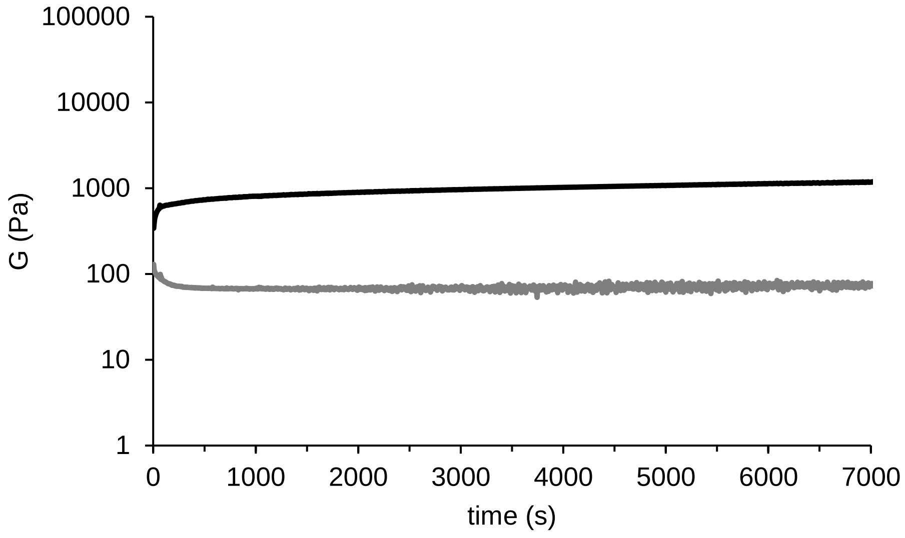

Fig. S6. Time sweep at 10 mM. Elastic modulus G' (black) and viscous modulus G'' (grey).

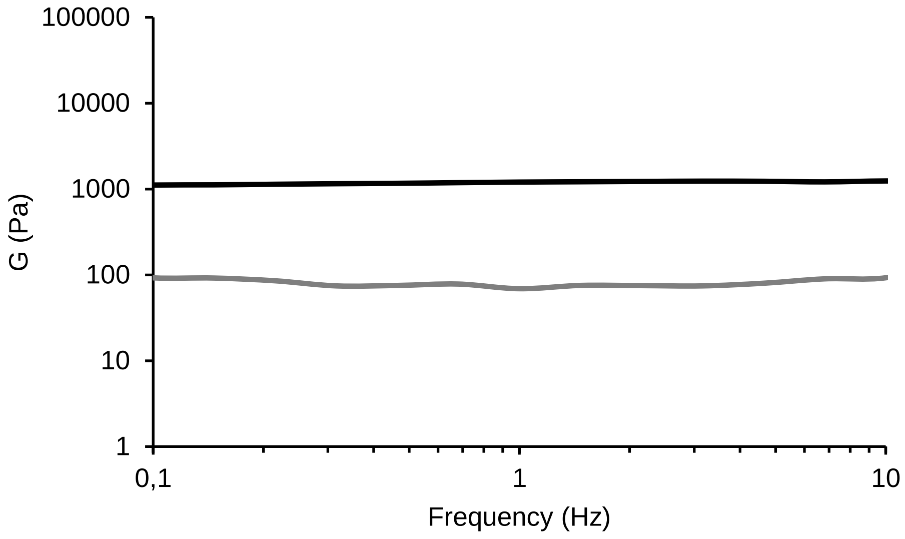

Fig. S7. Frequency sweep at 10 mM. Elastic modulus G' (black) and viscous modulus G'' (grey).

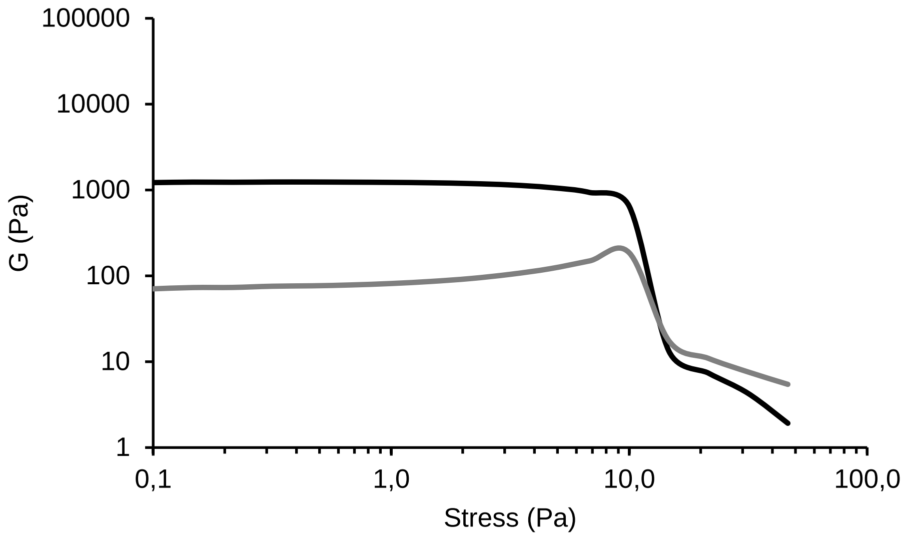

Fig. S8. Stress sweep at 10 mM. Elastic modulus G’ (black) and viscous modulus G’’ (grey).

3. Biocatalysis data

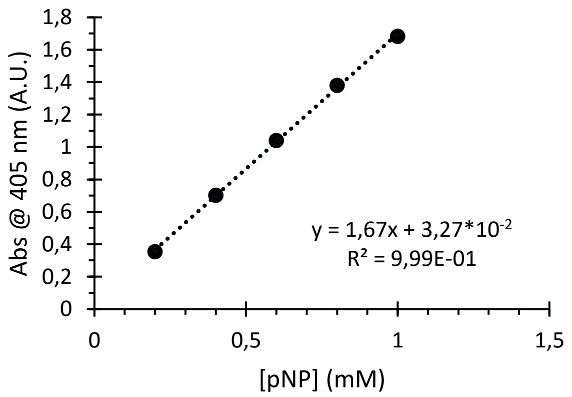

Fig. S9. Calibration curve for pNP.

[pNPA] (mM)	V_i (mM/s)
0.2	$6.25*10^{-6}$
0.4	$1.26*10^{-5}$
0.6	$1.87*10^{-5}$
0.8	$2.51*10^{-5}$
1.0	$3.13*10^{-5}$
1.2	$3.76*10^{-5}$
1.4	$4.38*10^{-5}$
1.6	$5.01*10^{-5}$
1.8	$5.63*10^{-5}$
2.0	$6.26*10^{-5}$

Fig. S10. Initial velocities (V_i) for blank reactions (without catalyst) in PBS at pH 7 (k_{obs} = $3.13*10^{-5}$).

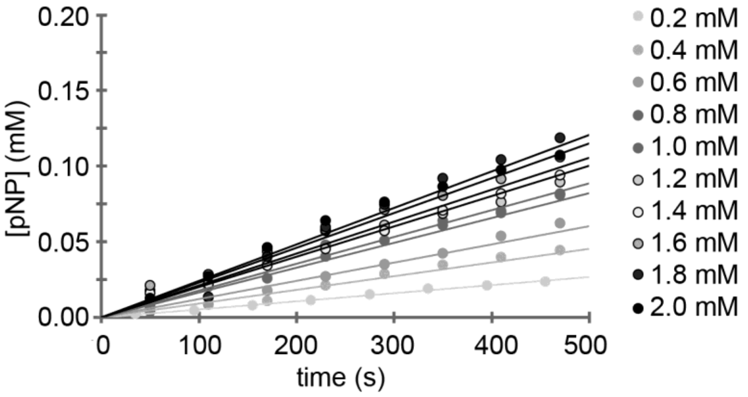

Fig. S11. Initial velocities (Vi) for the reaction with 0.1 mM peptide, which is the minimum concentration for significant catalysis to occur.

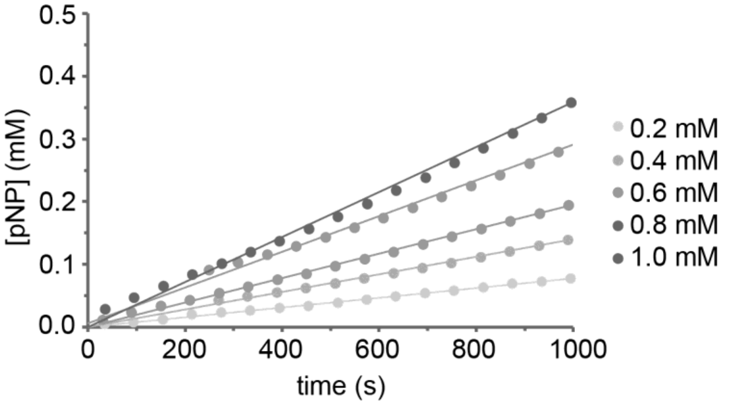

Fig. S12. Initial velocities (Vi) for the reaction with 1 mM peptide.

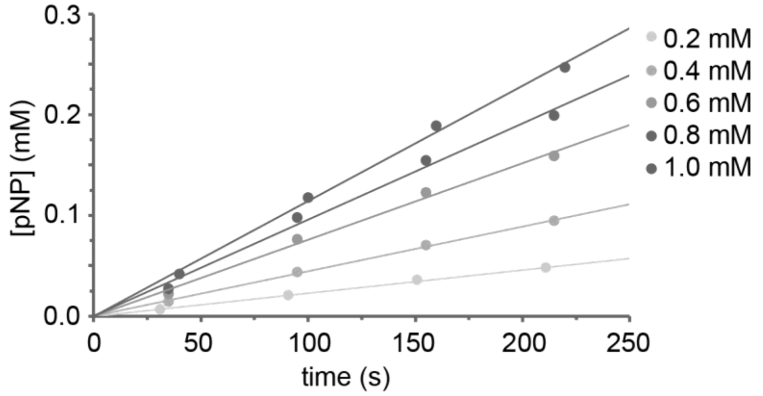

Fig. S13. Initial velocities (Vi) for the reaction with 10 mM peptide.
